# Controversies in the Surgical Management of Shoulder Instability: Open *vs* Arthroscopic Procedures

**DOI:** 10.2174/1874325001711010875

**Published:** 2017-08-31

**Authors:** Alejandro Huerta, Gustavo Rincón, Lluis Peidro, Andreu Combalia, Sergi Sastre

**Affiliations:** 1 Fellowship Universitat de Barcelona, Arthroscopy Department of Orthopaedic Surgery, Hospital Clínic, Barcelona, Spain; 2 Orthopedic Surgeon, Orthopedic Surgery Hospital Clínic Barcelona, Italy

**Keywords:** Shoulder dislocation, Shoulder instability, Open procedures, Arthroscopy

## Abstract

**Background:**

Traumatic anterior instability of the shoulder is a common condition associated with a high recurrence rate in young adults. Operative treatment reduces the risk of recurrence. Several studies have compared arthroscopic and open shoulder instability repair. The purpose of this paper is to perform a review of the literature where both techniques are compared in the repair of the anterior shoulder instability without bone loss.

**Methods:**

Prior to arthroscopy, recurrent dislocations were managed by open repair. There have been many studies documenting low recurrence rates after open Bankart stabilization.

Initially, arthroscopic fixation reported high failure rates.

**Results:**

In the last 20 years, the development of arthroscopic stabilization for recurrent anterior instability has improved failure rates. In comparison with open techniques, arthroscopic procedures have the advantages of decreased morbidity rate, early functional rehabilitation and improved range of motion.

**Conclusion:**

The available evidence does not show a statistically significant difference in outcome measures between arthroscopic and open repair for the treatment of recurrent anterior shoulder instability. Given the similar results between the 2 groups, differences in length of hospital stay and cost to the patient and society point to arthroscopic repair as the more judicious treatment approach.

## 1 INTRODUCTION

The shoulder is the most commonly dislocated major joint, with a reported incidence of 1.7% [1]. Symptomatic instability following dislocation is common, especially in young and active people [2]. Recurrent instability, occurring in 50% to 96% of patients who first dislocate under the age of 20 years and in 40% to 74% of patients between the ages of 20 and 40 years, limits range of movement of the joint, requires multiple hospital and emergency department admissions for treatment, and often calls for surgical procedures to prevent further dislocation [3-6]. Operative treatment reduces the risk of recurrence [7-9].

Several studies have compared arthroscopic and open shoulder instability repair. Proponents of the open technique have argued that it provides the surgeon the ability to provide a more anatomic and secure repair with improved anchor orientation, whereas proponents of the arthroscopic technique have contended that it avoids the complications associated with open approaches (infection, violation of the subscapularis, and arthrofibrosis) while providing an equivalent repair with a faster recovery [10].

Prior to arthroscopy, recurrent dislocations were managed by open repair. There have been many studies documenting low recurrence rates ranging from 0% to 11% after open Bankart stabilization [8, 11]. The classic open Bankart procedure includes incision of the subscapularis tendon and capsule to expose the anterior aspect of the labrum and capsule for secure reattachment to the glenoid rim with suture passed through bone tunnels. Multiple variations have been used to expose the joint, to repair the ligaments, and to treat associated capsular laxity.

The development of arthroscopic stabilization for recurrent anterior instability has undergone significant changes in the past 20 years. Initial arthroscopic fixation was performed by staple capsulorrhaphy, which resulted in recurrent instability in 16% to 33% of patients [12, 13]. Additional methods of arthroscopic stabilization have included transglenoid suturing, and bioabsorbable tack fixation, with a high failure rate. Newer techniques for arthroscopic stabilization have been developed, including suture anchor fixation and capsular plication, with failure rates decrease. Arthroscopic stabilization is the method preferred by most shoulder surgeon. Zhang *et al*. report that arthroscopic stabilization is performed in nearly 90% of shoulder stabilization surgeries and nearly doubled in incidence from 2004 to 2009 in the United States [14]

In the literature there are several retrospective comparative trials, case-control studies, randomized controlled trials and meta-analysis comparing both techniques with contradictory results.

The purpose of this paper is to perform a literature review of articles comparing open *versus* arthroscopic procedures of soft tissue. The following outcomes will be assessed separately: failure rate, complications, return to activity, cost and hospital stay.

## 2 FAILURE RATE

Initial arthroscopic fixation was performed by staple capsulorrhaphy, which resulted in recurrent instability in 16% to 33% of patients [12]. Posterior methods of arthroscopic stabilization have included transglenoid suturing, with a failure rate ranging from 0% to 49% [15-18] and bioabsorbable tack fixation, with a failure rate ranging from 9% to 23%. [16, 19-21]. In contrast, open approaches reported consistently low rates of recurrent instability [22-27].

Mothadi *et al*. in a meta-analysis that selected 11 studies concluded that evidence published through October 2003 shows that the open repair for recurrent traumatic anterior instability has a better outcome than arthroscopic repair with respect to recurrence and return to activity. This meta-analysis suggest that future randomized clinical trials are necessary to account for newer arthroscopic techniques [28].

Freedman, in six studies, found those 172 patients in the arthroscopic group (90 patients with transglenoid sutures, 77 patients with arthroscopic tacks, and 5 patients with suture anchors) and 156 patients in the open group. The groups were similar in demographic characteristics. When comparing the arthroscopic to the open group, there was a significantly higher rate of recurrent dislocation (12.6% *vs* 3.4%; P = .01) and total recurrence (recurrent dislocation or subluxation) (20.3% *vs* 10.3%; P = .01). In addition, there were a higher proportion of patients with an excellent or good postoperative Rowe score in the open group (88%) than in the arthroscopic group (71%) (P = .01). It also concluded that studies comparing open repair to new arthroscopic techniques using suture anchor fixation and capsular plication are necessary [16].

Lenters, reviewed 18 studies and found higher rates of recurrence with arthroscopic techniques [29].

In 2007, Hobby *et al*. in a review of the literature published between January 1985 and February 2006, reported that the failure rate of arthroscopic shoulder stabilization using staples or transglenoid suture techniques appeared to be significantly higher that of either open surgery or arthroscopic stabilization using suture anchors or bio-absorbable tacks. This is the first meta-analysis that arthroscopic anterior stabilization, using the most effective techniques, showed a similar rate of failure than open stabilization [30].

Petrera el al, in a meta-analysis that included only studies that compared open and arthroscopic repair using suture anchors. The rate of recurrent instability in the arthroscopic group was 6% *versus* 6.7% in the open group; rate of reoperation was 4.7% in the arthroscopic group *vs*. 6.6% in open (difference not statistically significant). They concluded that arthroscopic repair using suture anchors results in similar redislocation and reoperation rate compared to open Bankart repair [31].

A systematic review of level I and II trials between 1967 and 2010 showed no statistically differences in redislocation rates, return to activity, and functional outcomes between the arthroscopic and open repair groups. Range of motion was marginally better following arthroscopic treatment when compared with open repair. The major limitation of this review is that only 4 level I or II randomized controlled trials comparing arthroscopic and open surgical repair [32].

Recently systematic review of 8 overlapping meta-analyses comparing arthroscopic and open shoulder stabilization suggests that according to current best available evidence, there are no significant differences in failure rates [10].

In summary, although there is superiority in early studies of open surgery, however with new arthroscopic techniques, failure rates are similar

## 3 COMPLICATIONS

Proponents of the arthroscopic technique have contend that it avoids the complications associated with open approaches (infection, violation of the subscapularis, and arthrofibrosis). Green and Christensen report that arthroscopic stabilization procedures decreased operation-room time, blood loss, necrotic tissue, hospital stay, time for return to work and complications when compared with open procedures [33].

Pain, the studies available usually do not report the presence. Many studies report pain as a subset of the Rowe and Constant scores [39]. Persistent pain was reported with regard to treatment failure in the Bottoni *et al*. studies. One patient from the arthroscopic group had persistent shoulder pain without instability and was medically discharged from the military.

Open Bankart repair is associated with range of motion (ROM) restriction. Restrict external rotation and can lead to secondary osteoarthritis [4, 34-37]. Bottoni *et al*. reported the mean loss of motion (compared to the contralateral shoulder) was greater in the open shoulders. [8] Other studies noted a statistically significant improvement in ROM along several axes following arthroscopic surgery when compared with ROM following open repair. These differences were most notable in external rotation deficit [8, 38, 39]. Lenters *et al*. indicates that arthroscopic approaches are not as effective as open approaches in preventing recurrent instability or enabling patients to return to work. Arthroscopic approaches resulted in better function as reflected by the Rowe scores in the randomized clinical trials [29].

Loss of external rotation after open Bankart repair is an important prognostic factor for patient satisfaction. Rahme *et al*. demonstrated that open Bankart repair with a modified Rowe procedure is an excellent surgical option regarding stability, but restriction in external rotation reduces the likelihood of a satisfied patient [40].

Regarding to function of subescapularis muscle, there are several articles that inform this disjunction after a shoulder anterior approach, vertical and L-shaped tenotomy being the most damaging [41-43].

The biomechanical effect of placing a portal through the subscapularis tendon was studied for Khan *et al*. They demonstrated that penetrating the subscapularis tendon with either a 5-mm suture anchor or an 8-mm cannula does not produce any statistically significant change in strain compared with the native tendon [44].

In conclusion, arthroscopic repair offers advantages regarding range of motion and function of the subscapularis muscle compared with the open Bankart. There are no studies that directly evaluate the presence and intensity of pain.

## 4 RETURN TO ACTIVITY

Return to activity is a controversial item. There are studies with different results when both techniques are compared. Jorgensen *et al*. [38] employed a subjective patient survey to classify return to activity into 4 distinct groups. In the arthroscopic group, 18 patients (85.7%) regained good (7 patients, 33%) or excellent (11 patients, 52.4%) return to preinjury activity levels, while 2 patients (9.5%) were unchanged, and 1 patient (4.8%) suffered a reduction in activity level compared with pretreatment levels. Twenty patients (100%) treated by open technique in the Jorgensen trial regained activity, while 8 (40%) did so with some restrictions. Bottoni *et al*. defined return to pre-injury activity as full, active military duty without physical limitations and restrictions. Fifty-seven patients (93.4%) returned to preinjury activity levels, while 1 patient in the arthroscopic group reported persistent shoulder pain without instability and was discharged from the military [8].

Mohtadi *et al*. noted that open repair offers a better outcome than arthroscopic repairs with respect to return to activity. However, the inclusion of studies with a wide variety of study designs and a high risk of bias limits the reliability of these findings. Lenters *et al*. also found that open repair is superior with respect to postoperative functioning [28, 29].

Green reported that arthroscopic stabilization procedures decreased time for return to work when compared with open procedures [33].

## 5 COSTS AND HOSPITAL STAY

There are few articles comparing costs of arthroscopic *vs*. open shoulder stabilization. Wang *et al*. analyzed the patient outcomes, cost, and resource utilization of both procedures to determine if differences exist between open *versus* arthroscopic Bankart repair. Total operating room (OR) time (from time in the room to time exiting the room) and operating time (from skin incision to closure) were extracted from OR records for each case. Using the billing records for each case, OR charges were separated into time/personnel charges (OR service) and materials charges (OR equipment). Anesthesia charges were also extracted, and total charges for each case were then calculated, which included anesthesia and all OR charges, as well as admission and inpatient charges when applicable. They conclude that Arthroscopic Bankart repair has lower overall charges than open repair [45]. Green *et al*. attempted to quantitatively compare the operative time, and perioperative morbidity employing arthroscopic and open techniques. They reported that the arthroscopic Bankart procedure offers significant improvements in operative time, perioperative morbidity, and complications compared with the open technique for patients with anterior shoulder instability [33].

At this approach, arthroscopic techniques proved to be superior compared to open surgery.

## CONCLUSION

While the recurrent anterior shoulder instability has traditionally been treated by an open approach, more recently, arthroscopic techniques have achieved similar results.

In our institution we indicate arthroscopic procedure for the treatment of Bankart lesion. For Glenoid osseous defect less than 20-25%, we prefer arthroscopic repair; if the defect is greater we indicate Latarjet. For Engaging Hill-Sachs lesion we perform Remplissage. We believe that arthroscopy is a direct route to repair and restore a lesion that is anatomically intraarticular. It also allows to recognize and treat injuries that may be associated.

While limited, the available evidence from randomized controlled trials does not show a statistically significant difference in outcome measures between arthroscopic and open repair for the treatment of recurrent anterior shoulder instability. Specifically, studies comparing the currently accepted surgical techniques involving suture anchors did not show statistically significant differences in recurrence rates between the 2 treatment groups, however arthroscopic repair offers advantages regarding range of motion and function of the subscapularis muscle compared with the open Bankart. Given the similar results between the 2 groups, differences in length of hospital stay and cost to the patient and society point to arthroscopic repair as the more judicious treatment approach.
